# Mast Cells Express 11β-hydroxysteroid Dehydrogenase Type 1: A Role in Restraining Mast Cell Degranulation

**DOI:** 10.1371/journal.pone.0054640

**Published:** 2013-01-18

**Authors:** Agnes E. Coutinho, Jeremy K. Brown, Fu Yang, David G. Brownstein, Mohini Gray, Jonathan R. Seckl, John S. Savill, Karen E. Chapman

**Affiliations:** 1 University/British Heart Foundation Centre for Cardiovascular Research, University of Edinburgh, Edinburgh, United Kingdom; 2 Medical Research Council Centre for Inflammation Research, University of Edinburgh, Edinburgh, United Kingdom; 3 Mouse Pathology Core Laboratory, University of Edinburgh, Edinburgh, United Kingdom; 4 Medical Research Council Centre for Reproductive Health, University of Edinburgh, Edinburgh, United Kingdom; Fudan University, China

## Abstract

Mast cells are key initiators of allergic, anaphylactic and inflammatory reactions, producing mediators that affect vascular permeability, angiogenesis and fibrosis. Glucocorticoid pharmacotherapy reduces mast cell number, maturation and activation but effects at physiological levels are unknown. Within cells, glucocorticoid concentration is modulated by the 11β-hydroxysteroid dehydrogenases (11β-HSDs). Here we show expression and activity of 11β-HSD1, but not 11β-HSD2, in mouse mast cells with 11β-HSD activity only in the keto-reductase direction, regenerating active glucocorticoids (cortisol, corticosterone) from inert substrates (cortisone, 11-dehydrocorticosterone). Mast cells from 11β-HSD1-deficient mice show ultrastructural evidence of increased activation, including piecemeal degranulation and have a reduced threshold for IgG immune complex-induced mast cell degranulation. Consistent with reduced intracellular glucocorticoid action in mast cells, levels of carboxypeptidase A3 mRNA, a glucocorticoid-inducible mast cell-specific transcript, are lower in peritoneal cells from 11β-HSD1-deficient than control mice. These findings suggest that 11β-HSD1-generated glucocorticoids may tonically restrain mast cell degranulation, potentially influencing allergic, anaphylactic and inflammatory responses.

## Introduction

Mast cells play a central (typically initial) role in inflammatory and allergic reactions. They guard against bacterial pathogens and participate in tissue repair by producing mediators that promote vascular permeability, angiogenesis and fibrosis. Mast cells accumulate in chronically inflamed tissues in humans and in mice, and have consistently been observed in high numbers in human rheumatoid arthritis (reviewed, [1]), in Crohn’s disease (reviewed, [2]) and in the bronchus of asthmatic patients [3], [4]. Mast cells contain preformed tumour necrosis factor (TNF)-α in granules and can rapidly produce large amounts of both TNF-α and interleukin (IL)-1 [5] as well as other mediators, including histamine, eicosanoids (particularly prostaglandin D_2_) and vascular endothelial growth factor, which contribute to oedema, inflammation, hyperplasia and neovascularisation.

Glucocorticoids reduce mast cell number, maturation and activation [6], [7], [8], [9], contributing to the potent anti-allergic and anti-inflammatory effects of these steroids. Blood glucocorticoid levels depend upon activity of the hypothalamic-pituitary-adrenal axis. However, intracellular glucocorticoid concentrations can differ greatly from blood levels due to the action of 11β-hydroxysteroid dehydrogenase (11β-HSD), an enzyme that interconverts active glucocorticoids (cortisol in humans, corticosterone in rodents) and intrinsically inert 11-keto metabolites (cortisone, 11-dehydrocorticosterone). Two isozymes exist; 11β-HSD1 and 11β-HSD2. Whereas 11β-HSD2 inactivates glucocorticoids and is largely restricted to mineralocorticoid target tissues in the adult, 11β-HSD1 catalyses the opposite reaction *in vivo*, predominantly reactivating glucocorticoids by converting inert 11-keto-glucocorticoids into active forms and shows a more widespread distribution [10]. Mice homozygous for a targeted disruption of the *Hsd11b1* gene that encodes 11β-HSD1 (*Hsd11b1^−/−^* mice) have normal blood glucocorticoid levels on the C57BL/6J strain background [11] yet have a phenotype consistent with intracellular glucocorticoid deficiency (reviewed, [12]). Thus, they exhibit more severe acute inflammation in models of myocardial infarction, arthritis, sterile peritonitis and carageenan-induced pleurisy [13], [14]}, suggesting 11β-HSD1 normally exerts a restraining influence upon the early inflammatory response. *In vivo*, 11β-HSD1 expression is rapidly and markedly increased at sites of inflammation, including in peritoneal immune cells during sterile peritonitis [15], in colitis [16], [17] and in the arthritic joint [18]. 11β-HSD1 is expressed in macrophages [15], [19] where it performs an anti-inflammatory function, accelerating acquisition of macrophage phagocytic competence [15]). Expression has also been shown in dendritic cells [20], neutrophils [21] and lymphocytes [22], though its role in these cells remains uncharacterised. Here, we describe expression and activity of 11β-HSD1 in mast cells, classical glucocorticoid-targets in allergic and anaphylactic reactions, and demonstrate a restraining influence of 11β-HSD1 on mast cell degranulation.

## Materials and Methods

### Ethics Statement

All animal experimentation was conducted in strict accord with accepted standards of humane animal care under the auspices of the Animal (Scientific Procedures) Act UK 1986 following prior approval by the local University of Edinburgh ethical committee.

### Animals

Mice homozygous for a targeted disruption of the *Hsd11b1* gene on a C57BL/6J background (>8 backcrosses) have been described [15]. Control age-matched C57BL/6J (*Hsd11b1^+/+^*) mice were bred in-house. Mice were housed in groups of 2–5 per cage under controlled conditions (12 h-light/dark cycle, 21°C) with *ad libitum* access to water and standard rodent chow.

### Generation of Anti-glucose 6-phosphate Isomerase IgG Immune Complexes

Arthritogenic K/BxN serum containing anti-glucose 6-phosphate isomerase (GPI) IgG immune complexes was generated in house from arthritic K/BxN mice (expressing both the KRN T cell receptor transgene and the MHC class II molecule A^g7^) as described [14].

### Bone Marrow-derived (BMD) Mast Cell and Macrophage Cultures

BMD-mast cells and BMD-macrophages were cultured as previously described [15], [23] from 10 week old male C57BL/6 mice. Briefly, BMD-mast cells were obtained following 21d incubation in DMEM medium supplemented with recombinant mouse IL-3 (1 ng/ml) and SCF (50 ng/ml) (PeproTech EC Ltd, London, UK). Mast cell purity was confirmed by immunofluorescent staining with tryptase (mMCP-6) antibody and this protocol routinely gives >98% pure mast cells [24]. BMD-macrophages were obtained following 7d incubation in DMEM/F12 (Invitrogen, Paisley, UK) supplemented with 10% FCS, 500 U/ml penicillin, 500 U/ml streptomycin and 10% conditioned medium from murine fibrosarcoma cell (L929) cultures.

### Assay of 11β-HSD1 Activity

11β-HSD1 activity (dehydrogenase and reductase) was measured as previously described [15]. Briefly, 200 nM corticosterone or 11-dehydrocorticosterone, containing trace amounts of [^3^H]-corticosterone (specific activity ∼80 Ci/mmol; Amersham Pharmacia Biotech, Buckingham, UK) or [^3^H]-11-dehydrocorticosterone (made as previously described; [15]), was added to cell culture medium. At various times steroids were extracted in triplicate and analysed either by thin layer chromatography or by high performance liquid chromatography as previously described [15].

### RNA Extraction and Analysis

Total RNA was extracted from cells as previously described [15]. For RT-PCR, 1 µg RNA was reverse transcribed and subjected to PCR as described [15]. 11β-HSD2 primers: forward, 5′-CTGAAGCTGCTGCAGATTGGAT-3′ and reverse, 5′- GAGCAGCCAGGCTTGATAATG-3′. 11β-HSD1 PCR reactions all used a common reverse primer, 868P 5′- AGGATCCARAGCAAACTTGCTTGCA-3′, complementary to exon 6, and one of the following forward primers: 869P 5′-AAAGCTTGTCACWGGGGCCAGCAAA-3′, in exon 3, common to all 11β-HSD1 transcripts; ex2, 5′-GTCCCTGTTTGATGGCAG-3′, in exon 2, common to P1 and P2 transcripts; P1, 5′-GGAGCCGCACTTATCTGAA-3′, specific for transcripts arising from P1; P2, 5′-GGAGGTTGTAGAAAGCTCTG-3′, specific to transcripts arising from P2; P3, 5′-GTATGGAAAGCAAGACAAGG-3′, specific for transcripts arising from P3 (in the intron between exons 2 and 3). Real-time PCR was carried out on a LightCycler 480, according to the manufacturer’s instructions. Mastermix and primer-probe sets for 18S RNA (4331182), TATA-Binding Protein (TBP) (Mm00446973_m1), carboxypeptidase A3 (Mm00483940) and annexin 1 (Mm00440225_m1) cDNAs were purchased from Applied Biosystems (Warrington, UK). Neither internal standard differed between genotypes and accordingly TBP was used as the internal standard.

### Analysis of Peritoneal Mast Cells

For analysis of peritoneal mast cells, 8–12 week old male and female mice were used. Following peritoneal lavage, enriched peritoneal mast cells were prepared from freshly isolated peritoneal cells, positively selected for CD117^+^ mast cells using magnetic beads (Miltenyi Biotec, Surrey UK) according to the manufacturer’s instructions. Generally, around 50% pure mast cell populations were achieved based on histochemical staining of cytocentrifuge preparations or flow cytometry analysis. Mast cell degranulation was determined by measuring the release of β-hexosaminidase as described [25]. Briefly, peritoneal cells were incubated in triplicate for 15 min at 37°C in Tyrode’s Buffer and were untreated or treated with 10 µM ionomycin or diluted K/BxN serum. Following incubation, cells were collected by centrifugation. Aliquots (in triplicate) of the supernatant were transferred to a 96-well plate. The remaining supernatant was carefully removed and cell pellets solubilized in Tyrode’s buffer with the addition of 0.5% Triton X-100. Aliquots (in triplicate) of the solubilized pellet were also transferred to a 96-well plate. Next, β-hexosaminidase substrate (1 *p*-nitrophenyl-*N*-acetyl-b-D-glucosamine) was added to each well and incubated for 40 min at 37°C. Reactions were stopped by the addition of glycine and absorbance was measured at 405 nm. Total mast cell β-hexosaminidase was measured in untreated cells solubilized by the addition of Triton X-100. For each sample, degranulation was calculated as A_405_ supernatant/(A_405_ supernatant+A_405_ pellet). The mean of the untreated samples in each group was arbitrarily set to 100, and all other values expressed relative to this. Net degranulation was calculated as the difference between treated and untreated samples (and is thus % degranulation above levels in untreated cells). In preliminary experiments, no differences in mast cell number or level of degranulation were observed between the sexes of either genotype, therefore total peritoneal cells from both sexes were used.

### Flow Cytometry

Anti-mouse CD117 (c-Kit)-phycoerythrin (PE) or -allophycocyanin (APC), anti-mouse FcγR-PerCP Cy5.5, FcεR-PE and IgG controls (eBiosciences, Middlesex, UK) were added to peritoneal cells at concentrations recommend by the supplier and incubated on ice for 30 min in the dark. 11β-HSD1 sheep-derived antibody, generated in-house [26], was used in combination with Donkey anti-sheep secondary antibody (Alexa Fluor 488) (Invitrogen, Paisley, UK). During the staining procedure, cells were treated with a fixation and permeabilization kit (Fix and Perm, Invitrogen, Paisley, UK) according to the manufacturer’s instructions, in order to allow for intracellular staining with the 11β-HSD1 antibody. Fluorescence was determined by FACScalibur using Cellquest (Becton Dickinson UK Ltd, Oxford, UK) and analysed using FlowJo software (Treestar, Ashland, Oregon, USA).

### Transmission Electron Microscopy

Samples were fixed in 3% glutaraldehyde in 0.1M sodium cacodylate buffer, pH 7.3, for 2 h then washed 3 times (each for 10 min) in 0.1M sodium cacodylate. Specimens were post-fixed in 1% osmium tetroxide in 0.1M sodium cacodylate for 45 min, washed again (as above) then dehydrated in 50%, 70%, 90% and 100% acetone (10 min each), then twice further in 100% acetone. Samples were embedded in Araldite resin and 1 µm thick sections cut on a Reichert OMU4 ultramicrotome (Leica Microsystems UK Ltd, Milton Keynes, UK), stained with toluidine blue and viewed in a light microscope to select suitable areas for investigation. Ultrathin (60 nm thick) sections were cut from selected areas, stained in uranyl acetate and lead citrate then viewed in a Phillips CM120 Transmission electron microscope (FEI UK Ltd, Cambridge, UK). Images were captured using a Gatan Orius CCD camera (Gatan UK, Oxon, UK).

### Statistics

Student’s t-test was used for comparisons between genotypes. Significance was set at p<0.05. Values are means ± SEM.

## Results

### 11β-HSD1 is Expressed in Mast Cells

**Figure 1:**
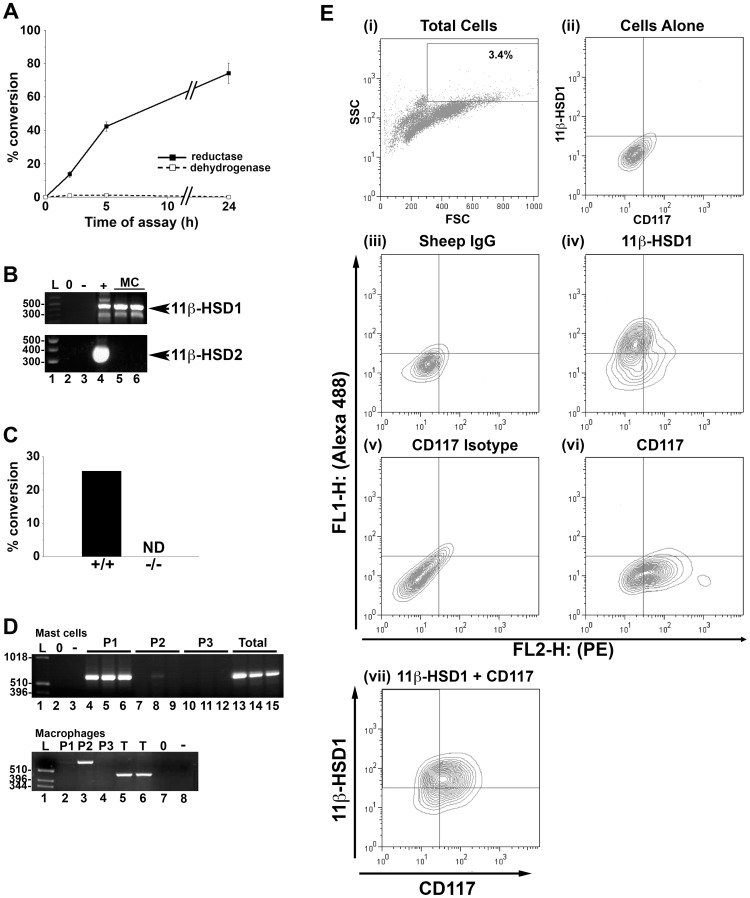
Mast cells express 11β-HSD1. (A) 11β-HSD1 reductase (conversion of 11-dehydrocorticosterone to corticosterone; Solid lines/black squares) and dehydrogenase (conversion of corticosterone to 11-dehydrocorticosterone; dashed lines/open squares) activities in BMD-MC (>98% pure, assessed by tryptase staining as previously described [24]) are expressed as % conversion of 200 nM substrate by 2×10^6^ cells, over time (h). Values are mean ± SEM of 3 independent assays carried out on pooled BMD-MC. (B) BMD-MC express 11β-HSD1 mRNA (upper panel; 469 bp RT-PCR product with primers 868P and 869P) but not 11β-HSD2 mRNA (lower panel; 400 bp RT-PCR product). Lanes 5 and 6 show RT-PCR products from 2 independent *Hsd11b1^+/+^* BMD-MC RNA samples. Positive controls (+) comprised liver mRNA (lane 4, upper panel) or kidney mRNA (lane 4, lower panel). Negative controls contained BMD-MC RNA but no reverse transcriptase (−, lane 3) or water (lane 2). Lane 1 contains a 100 bp ladder (Promega, Southampton, UK). (C) *Hsd11b1^−/−^* BMD-MC lack 11β-HSD1 activity. 11β-HSD1 reductase activity was measured in *Hsd11b1^+/+^* (+/+) and *Hsd11b1^−/−^* (−/−) BMD-MC. Data are expressed as % conversion of 200 nM 11-dehydrocorticosterone to corticosterone by 2×10^6^ cells in a 10 h assay. Values are means of triplicate assays performed on pooled BMD-MC. ND, not detected. (D) Mast cells transcribe *Hsd11b1* from the P1 promoter, whereas macrophages use the P2 promoter. Upper panel; RT-PCR products from 3 independent BMD-MC RNA samples showing the 627 bp RT-PCR product from P1 transcripts of 11β-HSD1 (lanes 4–6), but not P2 (predicted product of 647 bp, lanes 7–9) or P3 (predicted product of 542 bp, lanes 10–12). Kidney RNA confirmed transcription from P3 (not shown). Total 11β-HSD1 mRNA was detected using ex2 and 868P primers common to all transcripts (lanes 13–15, 587 bp product). Lower panel; RT-PCR products from BMD macrophage RNA showing the 647 bp RT-PCR P2 product (lane 3), but not the P1 (lane 2) or P3 products (lane 4). Total 11β-HSD1 mRNA (T) was detected using 868P and 869P primers, common to all transcripts (lanes 5, 6; 469 bp product). Lane 1 contains a 1 kb ladder (Invitrogen, Paisley, UK). Lanes marked (0) contain water only and lanes marked (−) show RT-PCR reactions from which the RT was omitted. (E) Flow cytometric staining revealed 11β-HSD1^+^CD117^+^ peritoneal cells. Freshly isolated peritoneal cells (∼5×10^5^ cells per sample) from male C57BL/6 mice were stained with 11β-HSD1 and CD117^+^ antibodies. Total peritoneal cells were first gated according to side scatter (SSC) and forward scatter (FSC) (i), then cells with high granularity, where mast cells lie (see Figure S1), were assessed for 11β-HSD1 and CD117 staining (ii–vii). Controls included; (ii) high SSC cells alone, (iii) sheep IgG control for 11β-HSD1 antibody, (iv) 11β-HSD1 antibody only, (v) isotype control for CD117 and (vi) CD117 antibody only. Panel (vii) shows cells double stained with 11β-HSD1 and CD117 antibodies.

With 11-dehydrocorticosterone as substrate, BMD-mast cells show 11β-keto-reductase activity (Figure 1A; 16.9±1.1 pmol corticosterone/h/10^6^ cells). In contrast, BMD-mast cells show negligible 11β-dehydrogenase activity with corticosterone as substrate (Figure 1A) showing the absence of 11β-HSD2 and indicating 11β-HSD1 is a predominant 11β-reductase in mast cells. Consistent with this, 11β-HSD1 mRNA is present and 11β-HSD2 mRNA absent in mast cells (Figure 1B). BMD-mast cells from 11β-HSD1-deficient mice lack 11β-HSD1 activity (Figure 1C), confirming the activity is due to 11β-HSD1. 11β-HSD1 mRNA is transcribed from 3 distinct promoters [27], [28]. Transcription of the *Hsd11b1* gene in BMD-mast cells initiates at the upstream P1 promoter, with little or no transcription from the downstream P2 and P3 promoters (Figure 1D). Importantly, this contrasts with BMD-macrophages which exploit the P2 promoter (Figure 1D), suggesting that these P1-initiated transcripts are in mast cells and not in contaminating macrophages. CD117 or c-Kit, is a marker for mature mast cells. Flow cytometric staining of lavaged peritoneal cells (of which ∼3% are mast cells; see Figure S1 for definition of the CD117^+^ population) with 11β-HSD1 antibody demonstrates immunoreactive protein in CD117^+^ cells (Figure 1E), confirming 11β-HSD1 expression in mast cells *in vivo* as well as *in vitro*.

### 11β-HSD1-deficiency Alters Peritoneal Mast Cell Number and Phenotype

**Figure 2:**
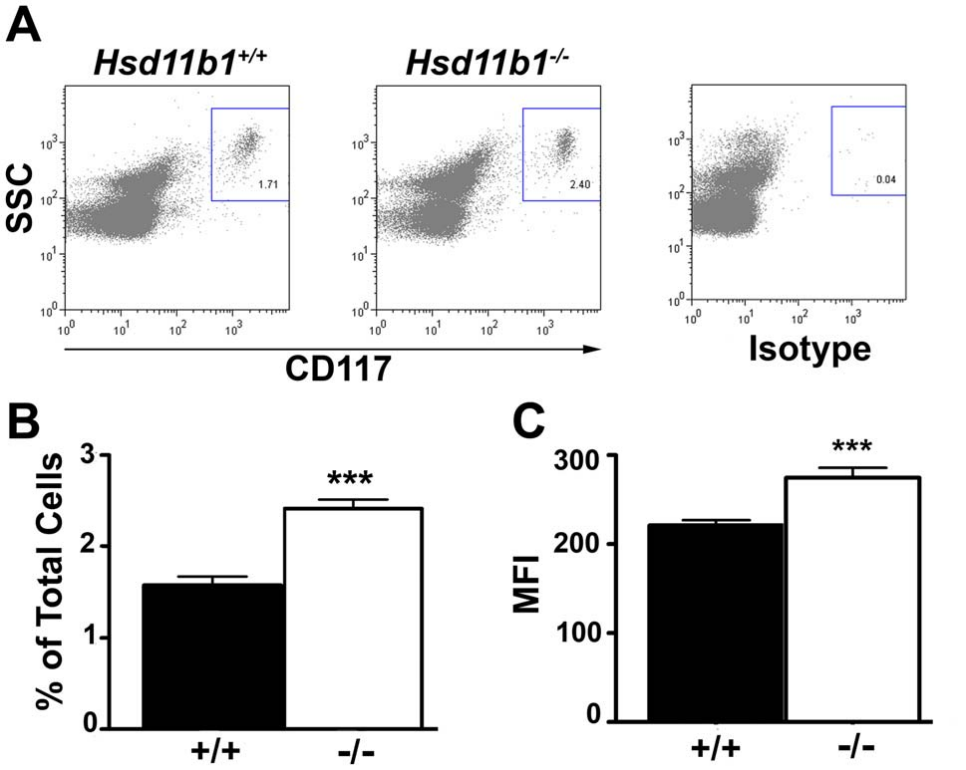
More mast cells in the peritoneum of Hsd11b1^−/−^ mice. (A) Representative dot plots of freshly isolated peritoneal cells (5×10^5^) from *Hsd11b1^+/+^* (left) and *Hsd11b1^−/−^* (middle) mice stained for CD117 (a mast cell marker) or isotype control (right) and subject to flow cytometry. (B) Peritoneal cells from *Hsd11b1^−/−^* mice (−/−, white bars) have more CD117^+^ cells than *Hsd11b1^+/+^* (+/+, black bars), expressed as percentage of total cells (total cell number; *Hsd11b1^+/+^*, 3.8±0.5×10^6^ *vs Hsd11b1^−/−^*, 3.7±0.5×10^6^, p>0.05). (C) CD117^+^ cells from *Hsd11b1^−/−^* mice have higher mean fluorescence intensity (MFI) compared to *Hsd11b1^+/+^* mice (black bars). Data are mean ± SEM, n = 8, *p<0.05, **p<0.01.

Mast cell number and phenotype are both influenced by glucocorticoids [6], [7], [8], [9]. To address whether either are affected by 11β-HSD1-deficiency, peritoneal mast cells from naive *Hsd11b1^−/−^* mice were subject to flow cytometry. More CD117^+^ cells are present in the peritoneum of naïve *Hsd11b1^−/−^* mice than controls (Figure 2A, B) and CD117^+^ cells from *Hsd11b1^−/−^* mice have higher mean fluorescence intensity (Figure 2C) suggesting increased surface expression of CD117 on peritoneal mast cells of *Hsd11b1^−/−^* mice. However, the proportion of CD117^+^ cells also positive for FcεR and FcγR mast cell markers is the same in both genotypes (96.8±0.4% of *Hsd11b1^+/+^* and 96.9±0.9% of *Hsd11b1^−/−^* CD117^+^ cells express both FcεR and FcγR markers) with no difference in mean fluorescence intensity of these receptors between genotypes (FcεR-PE mean fluorescence intensity; *Hsd11b1^+/+^*31.2±0.5 *vs Hsd11b1^−/−^,* 28.3±2.0 and FcγR-PerCP Cy5.5 mean fluorescence intensity; *Hsd11b1^+/+^,* 51.5±2.0 *vs Hsd11b1^−/−^,* 53.9±0.5).

**Figure 3:**
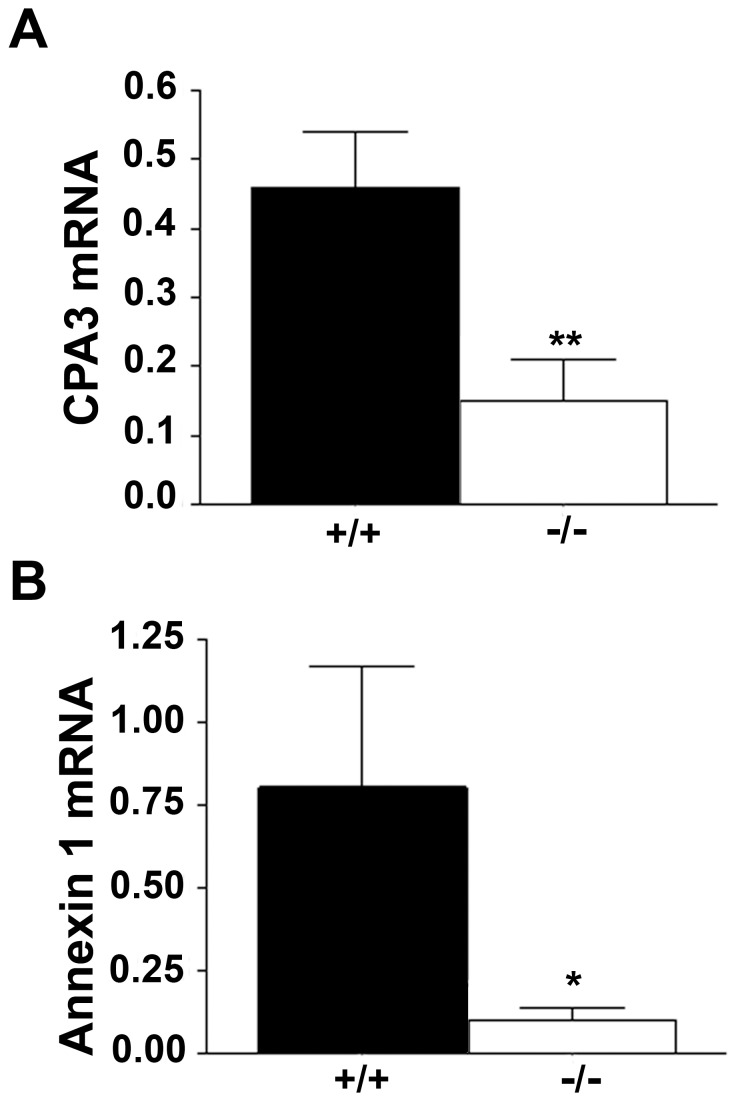
Decreased levels of glucocorticoid-sensitive transcripts in peritoneal cells from Hsd11b1^−/−^ mice. Real-time PCR measurement of (A) Carboxypeptidase A3 and (B) annexin 1 mRNA levels in total peritoneal cells from naïve male *Hsd11b1^−/−^* (−/−; white bars) or *Hsd11b1^+/+^* (+/+; black bars) mice. Data are mean ± SEM; n = 8, *p<0.05, **p<0.01. CPA; carboxypeptidase A3.

To determine whether mast cell contents are affected by 11β-HSD1 deficiency, key glucocorticoid-regulated transcripts were examined. Levels of mRNA encoding carboxypeptidase A3, generally considered a mast cell-specific product [29], [30] that is glucocorticoid-inducible [8], are lower in peritoneal cells from *Hsd11b1^−/−^* mice, compared to controls despite increased peritoneal mast cell number, consistent with reduced intracellular glucocorticoid action in 11β-HSD1-deficient mast cells (Figure 3A). Moreover, although not mast cell-specific, levels of annexin I mRNA (formerly called lipocortin), a classic glucocorticoid-inducible gene [31], are also reduced in peritoneal cells from *Hsd11b1^−/−^* mice (Figure 3B).

**Figure 4:**
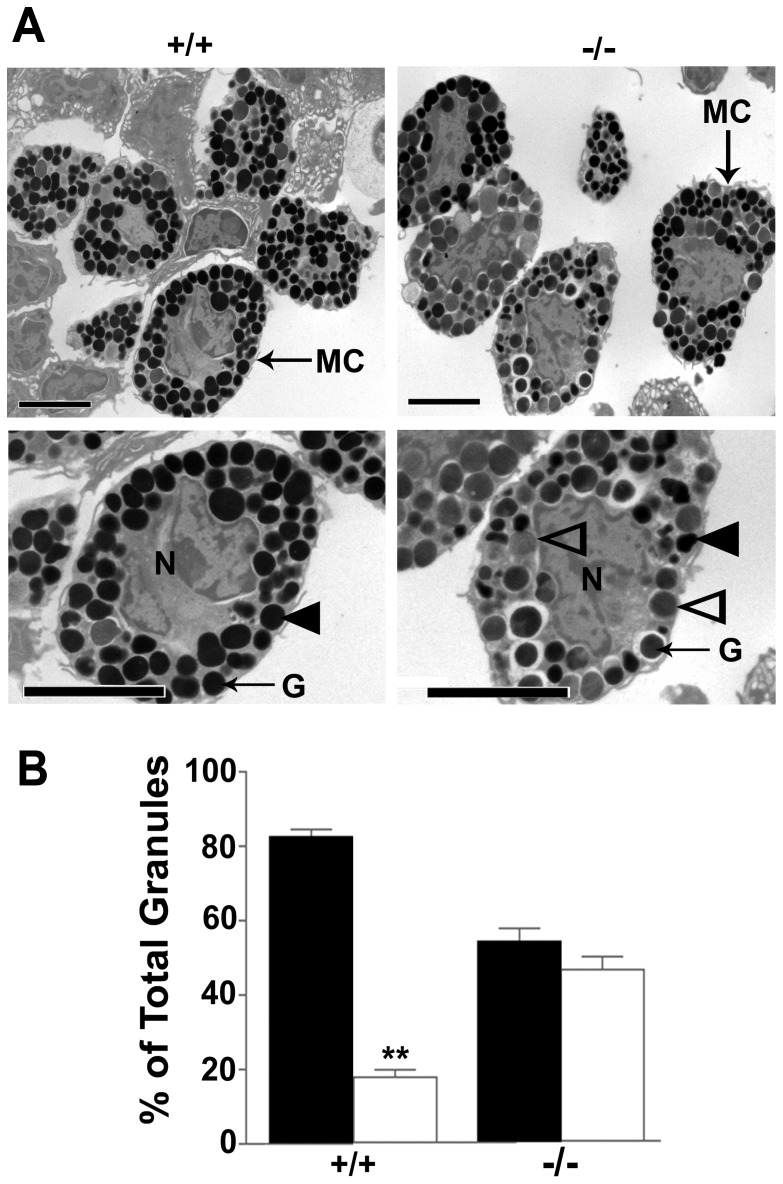
Ultrastructural analysis suggests piecemeal degranulation in Hsd11b1^−/−^ mast cells. (A) Representative EM images showing peritoneal mast cells from *Hsd11b1^−/−^* (−/−) and *Hsd11b1^+/+^* (+/+) mice. Closed arrowheads indicate dark (dense) granules and open arrowheads indicate lighter (less dense) granules. The lighter granules are suggestive of piecemeal degranulation in *Hsd11b1^−/−^* mice. MC; mast cell, G; granule, N; nucleus. (B) Peritoneal mast cells from *Hsd11b1^−/−^* (−/−) mice have fewer electron dense granules (black bars) and more “lighter” granules (white bars) compared to mast cells from *Hsd11b1^+/+^* (+/+) mice. At least 40 individual mast cells from a pooled sample from each genotype were scored by 2 independent observers, blind to genotype. Data are expressed as mean ± SEM; **p<0.01.

To investigate whether the characteristic granule morphology of mast cells is affected by 11β-HSD1-deficiency, transmission electron microscopy was carried out on an enriched population of peritoneal mast cells (∼50% mast cells). Total granule number per cell did not differ between genotypes *Hsd11b1^+/+^*55.6±14.7 (n = 41) *vs Hsd11b1^−/−^*61.6±19.4 (n = 49). However, whereas mast cells from control mice predominantly contain homogeneously electron dense granules (Figure 4A), those from *Hsd11b1^−/−^* mice are more heterogeneous and contain a greater proportion of lighter granules (Figure 4A, B) as well as enlarged partially filled and empty vesicles, characteristic of piecemeal degranulation [32], a slower form of mast cell degranulation than anaphylactic degranulation, that has been associated with persistent inflammation [32].

### 11ß-HSD1 Deficiency Reduces the Activation Threshold of Peritoneal Mast Cells

**Figure 5:**
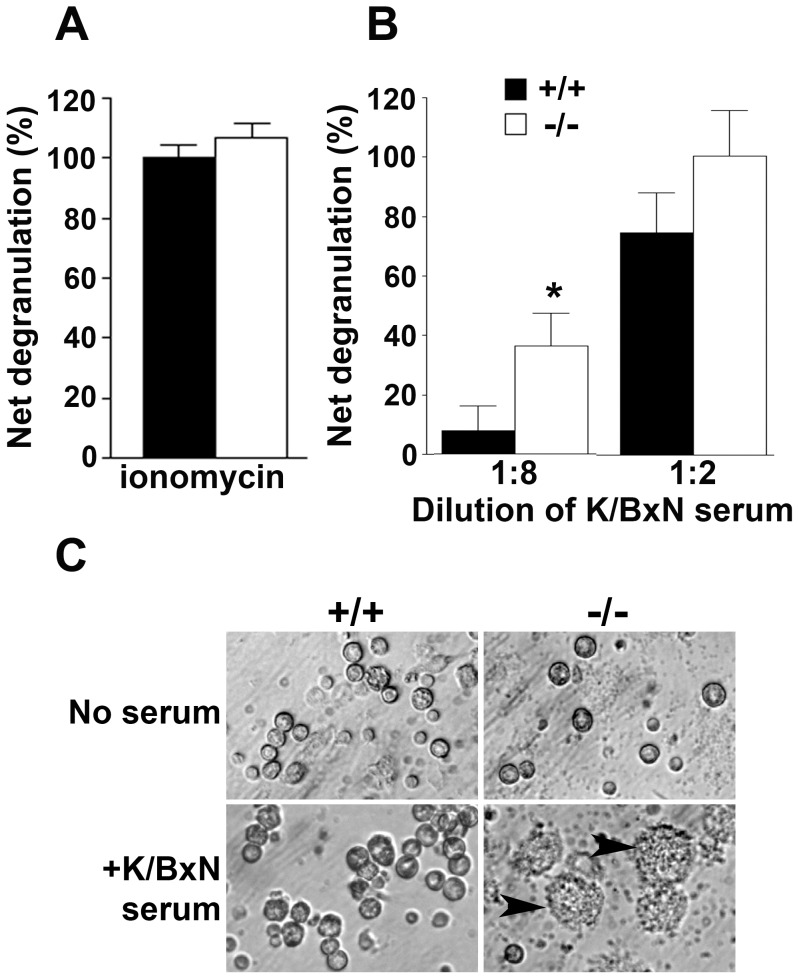
Hsd11b1^−/−^ peritoneal mast cells are hyper-responsive to degranulation induced by K/BxN serum. Release of β-hexosaminidase from peritoneal cells (2×10^6^) was measured following 15 min incubation with either (A) 10 µM ionomycin or (B) K/BxN serum (diluted 1∶8 or 1∶2 in Tyrode’s buffer). Black bars, *Hsd11b1^+/+^*; white bars, *Hsd11b1^−/−^*. Data are net degranulation in treated cells above levels measured in untreated cells (cells only are set to zero; see methods for details), and are mean ± SEM; n = 12−13, *p<0.05. (C) Representative micrographs (captured at 40× magnification) of enriched peritoneal CD117^+^ cells from *Hsd11b1^+/+^* (+/+, left panels) or *Hsd11b1^−/−^* mice (−/−, right panels) following 21 h incubation with buffer (top panels) or K/BxN serum (1∶8 dilution) (lower panels). Arrowheads indicate degranulated mast cells.

To determine whether 11β-HSD1-deficiency functionally alters mast cells as suggested by the granule morphology, we measured mast cell degranulation by assaying β-hexosaminidase release. Maximal degranulation induced by 10 µM ionomycin was similar in peritoneal cells from *Hsd11b1^−/−^* and control mice (Figure 5A). A similar maximal level of degranulation was observed following 15 min incubation with a 1∶2 dilution of K/BxN serum (containing anti-glucose 6-phosphate isomerase IgG immune complexes, a potent trigger of mast cell degranulation) into culture medium (Figure 5B). However, 15 min incubation with a lower concentration of K/BxN serum (diluted 1∶8 in medium) induced significantly more degranulation of peritoneal mast cells from *Hsd11b1^−/−^* than control mice (Figure 5B). Furthermore, when an enriched population of peritoneal CD117^+^ cells (∼50% mast cells) was incubated for 21 h with K/BxN serum (diluted 1∶8 in medium), microscopic examination revealed extensive degranulation of 11β-HSD1-deficient mast cells, whereas controls showed little or no degranulation (Figure 5C). 11β-HSD1-deficiency therefore lowers the threshold for degranulation of resident mast cells following IgG immune complex activation.

## Discussion

Pharmacological levels of glucocorticoids have potent effects on mast cells, but effects of endogenous glucocorticoids at physiological levels are unknown. Mast cells from *Hsd11b1^−/−^* mice have a phenotype consistent with relative glucocorticoid deficiency, including reduced expression of glucocorticoid-sensitive mast cell-specific carboxypeptidase A3, suggesting 11β-HSD1-mediated glucocorticoid amplification tonically suppresses mast cell responses.

11β-HSD1 is expressed in both BMD- and in peritoneal mast cells. At around 10–20 pmol/h/10^6^ cells, the level of keto-reductase activity in BMD-mast cells is considerably higher than that reported for T lymphocytes (<0.1 pmol/h/10^6^ cells; [22]) and comparable with levels in immature BMD-dendritic cells (7 pmol/h/10^6^ cells; [20]) and BMD-macrophages (12 pmol/h/10^6^ cells). Thus, the measured activity is likely to be due to mast cells and not to contamination with macrophages or another cell type expressing 11β-HSD1, present at <2% of the population. This is supported by the use of the P1 promoter in mast cells, which contrasts with most other tissues and cell types, including macrophages, that use the P2 promoter and suggests a distinct regulation in mast cells.

Whilst the maximum response to degranulating stimuli was similar in mast cells from *Hsd11b1^−/−^* and control mice (suggesting *Hsd11b1^−/−^* mice have normal levels of β-hexosaminidase in stored granules), 11β-HSD1-deficient cells were sensitive to a low dose of K/BxN serum that was ineffective in control cells, suggesting that it is the threshold for activation that is reduced, rather than the total number of stored granules, which was unchanged in *Hsd11b1^−/−^* mice. Ultrastructural analysis of peritoneal mast cells supported this “trigger-happy” phenotype of *Hsd11b1^−/−^* mast cells and indicated piecemeal degranulation, a form of non-anaphylactic granule release observed in mast cells *in situ*, possibly representing up-regulated constitutive secretion [32]. This increased sensitivity is likely to reflect a difference in differentiation, maturation or activation of mast cells, as it is preserved in the absence of 11β-HSD1 substrate, *ex vivo*. Density of FcγR, key for IgG-mediated signalling, is normal on 11β-HSD1-deficient mast cells, though it is possible that the threshold for FcγR activation and signalling is reduced. Mast cell number was increased by ∼34% in the peritoneum of *Hsd11b1^−/−^* mice. Mast cell number is reduced by pharmacological glucocorticoid treatment *in vivo*, though whether normal physiological glucocorticoid concentrations have a similar effect *in vivo* has not been reported. However, given that *Hsd11b1^−/−^* mice have normal plasma corticosterone levels on this genetic background [33] and we have previously shown normal responses of *Hsd11b1^−/−^* immune cells to exogenous corticosterone [15], the increase in the number of mast cells in the peritoneum of *Hsd11b1^−/−^* mice suggests that 11β-HSD1-mediated regeneration of endogenous intracellular glucocorticoids limits proliferation of at least some mast cell populations. In this respect, it is interesting that activation products of mast cells are chemoattractants for their progenitors [34], [35], suggesting that the basal activation observed in *Hsd11b1^−/−^* mast cells may underlie the increase in peritoneal mast cell number.

Immune activation is a potent stimulus to the hypothalamic-pituitary-adrenal (HPA) axis and removal of endogenous glucocorticoids or blockade of their actions exacerbates immune and inflammatory disease in humans and in animal models (reviewed, [36]). Our data suggest that by providing a “brake” to spontaneous mast cell degranulation, 11β-HSD1-mediated amplification of endogenous glucocorticoid action within mast cells may influence allergic and anaphylactic reactions, in which mast cells are central, and for which glucocorticoid phamacotherapy remains a highly effective treatment. Experimental testing of this hypothesis in appropriate *in vivo* models of anaphylaxis and allergy will be important in the future. These will be important considerations in the clinical introduction of selective 11β-HSD1 inhibitors for treatment of metabolic disease, a side-effect of which could be mast cell hyper-responsiveness.

## Acknowledgments

We thank Steve Mitchell for carrying out the electron microscopy, Pamela Knight for providing additional BMD-mast cell cDNA, Tiina Kipari for advice and assistance with flow cytometry analysis, staff of the BRR and Spike Clay for expert animal assistance and colleagues, especially Alan Pemberton and Hugh Miller, for helpful discussions.

## Supporting Information

Figure S1 **Flow cytometric assessment of mast cells using an enriched population of CD117^+^ peritoneal cells.** Freshly obtained total peritoneal cells (3.8×10^7^ cells pooled from 6 mice) were labeled with CD117 magnetic beads and purified using a MACS magnet. (A) Using flow cytometry separate populations; total cells (i–ii), CD117^+^ (iii–iv) and CD117^−^ (v–vi) cells, were assessed for mast cells using side scatter (SSC), forward scatter (FSC) and staining with CD117 antibody. Panels (i, iii, v) illustrate the gate for high SSC/CD117^+^ cells, while panels (ii, iv, vi) illustrate where the high SSC/CD117^+^ cells (black) are positioned against all cells in the sample (grey). This gate was then used to select for mast cells in all experiments. (B) Mast cells were confirmed by staining the CD117^+^ enriched-high SSC/CD117^+^ population of cells (A (iii) top right gate) with Fc Gamma receptor (Fc Gamma R) and Fc Epsilon receptor (Fc Epsilon R) (markers of mast cells) antibodies (iii). Panel (i) shows single stain control for CD117 only, in the high SSC/CD117^+^ population of cells, negative for Fc Gamma R and Fc Epsilon R staining.
(TIF)
