# Supplementary figures and images for: Mast Cells Express 11β-hydroxysteroid Dehydrogenase Type 1: A Role in Restraining Mast Cell Degranulation
Source: PLoS One. 2013 Jan 18;8(1):e54640. doi: 10.1371/journal.pone.0054640 (PMC3548897; doi:10.1371/journal.pone.0054640)

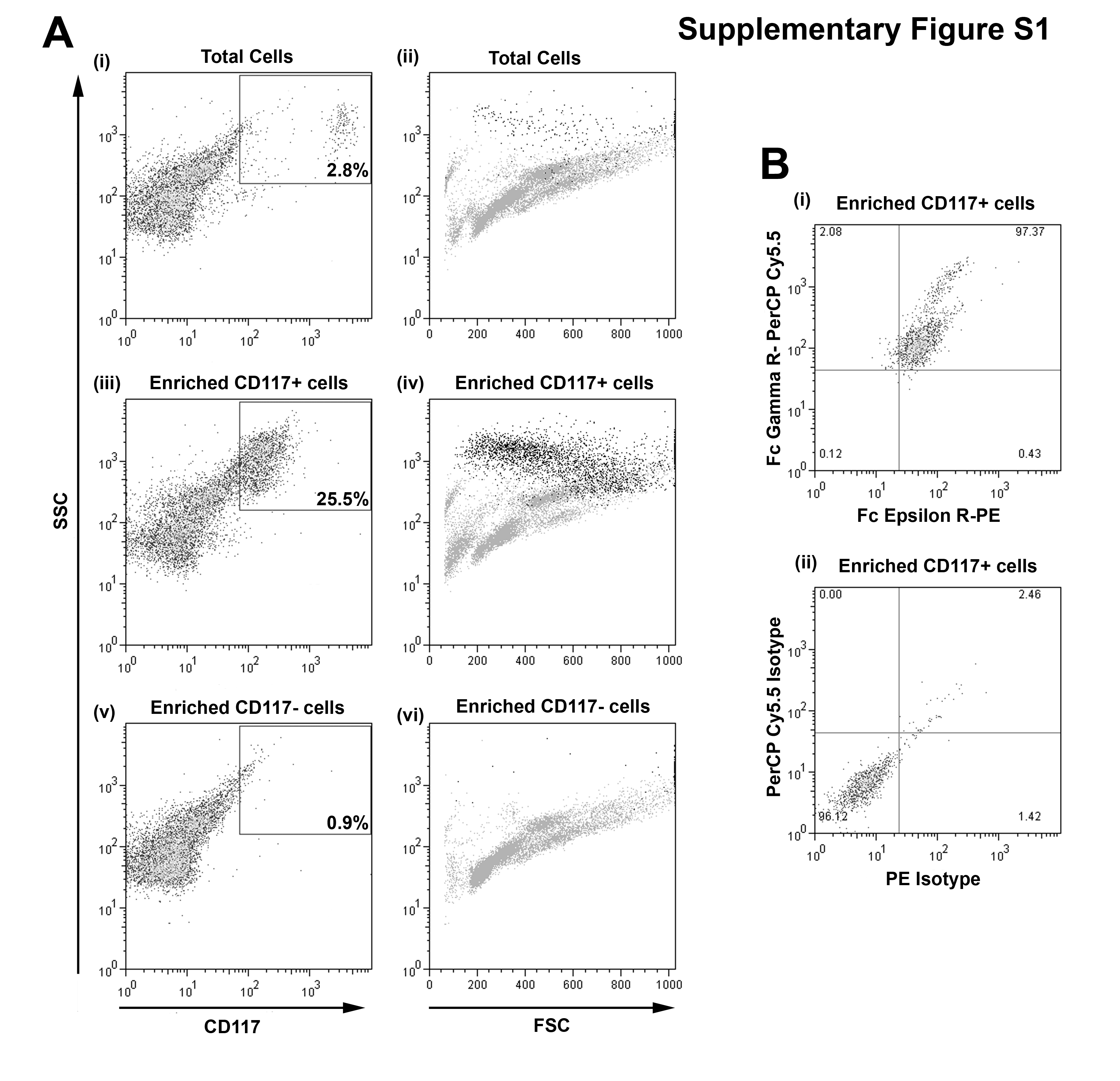

Supplement: Figure S1 — Flow cytometric assessment of mast cells using an enriched population of CD117+ peritoneal cells. Freshly obtained total peritoneal cells (3.8×10^7^ cells pooled from 6 mice) were labeled with CD117 magnetic beads and purified using a MACS magnet. (A) Using flow cytometry separate populations; total cells (i–ii), CD117+ (iii–iv) and CD117− (v–vi) cells, were assessed for mast cells using side scatter (SSC), forward scatter (FSC) and staining with CD117 antibody. Panels (i, iii, v) illustrate the gate for high SSC/CD117+ cells, while panels (ii, iv, vi) illustrate where the high SSC/CD117+ cells (black) are positioned against all cells in the sample (grey). This gate was then used to select for mast cells in all experiments. (B) Mast cells were confirmed by staining the CD117+ enriched-high SSC/CD117+ population of cells (A (iii) top right gate) with Fc Gamma receptor (Fc Gamma R) and Fc Epsilon receptor (Fc Epsilon R) (markers of mast cells) antibodies (iii). Panel (i) shows single stain control for CD117 only, in the high SSC/CD117+ population of cells, negative for Fc Gamma R and Fc Epsilon R staining. (TIF) [file pone.0054640.s001.tif]
